# Use and perceived added value of patient-reported measurement instruments by physiotherapists treating acute low back pain: *a survey study among Dutch physiotherapists*

**DOI:** 10.1186/s12891-020-3132-9

**Published:** 2020-02-24

**Authors:** J. Knoop, W. van Lankveld, F. J. B. Geerdink, R. Soer, J. B. Staal

**Affiliations:** 1 HAN University of Applied Sciences, Musculoskeletal Rehabilitation research group, Nijmegen, the Netherlands; 2 Saxion University of Applied Sciences, Enschede, the Netherlands; 3 University Medical Center Groningen, Groningen Spine Center, University of Groningen, Groningen, the Netherlands; 4 Radboud Institute for Health Sciences, IQ Healthcare, Radboud University Medical Centre, Nijmegen, the Netherlands

**Keywords:** Low back pain, Physiotherapy, Measurement instrument, Screening tool, Patient-reported outcome measurement, Survey study

## Abstract

**Background:**

This study aims to explore (i) physiotherapists’ current use in daily practice of patient-reported measurement instruments (screening tools and questionnaires) for patients with acute low back pain (LBP), (ii) the underlying reasons for using these instruments, (iii) their perceived influence on clinical decision-making, and (iv) the association with physiotherapist characteristics (gender, physiotherapy experience, LBP experience, overall e-health affinity).

**Methods:**

Survey study among Dutch physiotherapists in a primary care setting. A sample of 650 physiotherapists recruited from LBP-related and regional primary care networks received the survey between November 2018 and January 2019, of which 85 (13%) completed it.

**Results:**

Nearly all responding physiotherapists (98%) reported using screening tools or other measurement instruments in cases of acute LBP; the Quebec Back Pain Disability Scale (64%) and the STarT Back Screening Tool (61%) are used most frequently. These instruments are primarily used to evaluate treatment effect (53%) or assess symptoms (51%); only 35% of the respondents mentioned a prognostic purpose. Almost three-quarters (72%) reported that the instrument only minimally impacted their clinical decision-making in cases of acute LBP.

**Conclusions:**

Our survey indicates that physiotherapists frequently use patient-reported measurement instruments in cases of acute LBP, but mostly for non-prognostic reasons. Moreover, physiotherapists seem to feel that current instruments have limited added value for clinical decision-making. Possibly, a new measurement instrument (e.g., screening tool) needs to be developed that does fit the physiotherapist’s needs and preferences. Our findings also suggest that physiotherapist may need to be more critical about which measurement instrument they use and for which purpose.

## Background

Non-specific low back pain (LBP) is one of the major global health problems More than any other musculoskeletal disorder, it is responsible for the most years lived with disability [1]. The costs of care, investigations and lost productivity associated with LBP are also a significant societal burden [2]. Almost everyone will experience at least a short episode of LBP during their lifetime [3]. Patients with LBP are the most prevalent patient group consulting a primary care physiotherapist in the Netherlands [4]. Moreover, around half of the patients consulting a physiotherapist do not visit a general practitioner prior to their physiotherapy consult, leaving the physiotherapist to play not only an important role in treatment, but also in the diagnosis and prognosis of LBP [5].

Most people who suffer an acute episode of non-specific LBP will recover in a few weeks or months with no or only minimal treatment, whereas around 10 to 25% will develop chronic LBP (i.e. pain lasting longer than three months) [3, 6]. Chronic LBP is the most problematic type of LBP because its prognosis is very poor [7], it accounts for the majority of costs [8, 9] and the effects of interventions are small [10]. It is highly relevant to distinguish between patients who are expected to have a favourable or unfavourable course of LBP symptoms (i.e. those who are likely to become chronic) as soon as possible [11, 12]. Patients with an expected favourable course of LBP should be reassured and given only minimal care at most [13, 14], thereby avoiding unnecessary care and its associated costs [15]. For patients with an expected unfavourable course of LBP, physiotherapists are recommended to provide treatment (primarily patient education and exercise therapy) or to refer patients to specialised professionals and/or multidisciplinary care [13]. Early targeted treatment of at-risk patients might prevent chronification of the complaints while reducing costs of care and lifelong suffering. Such a stratification of care has been found to improve the overall effectiveness of LBP treatment in a cost-effective manner [16].

A number of prognostic screening tools are available for physiotherapists. For instance, the Orebro Musculoskeletal Pain Questionnaire (OMPQ) [17] and the STarT Back Screening Tool (SBT) [18, 19] have been developed and validated to estimate risk in LBP. Their use is recommended in physiotherapy guidelines from New Zealand [20] and England [21]. The Dutch physiotherapy guideline [13] does not recommend any specific screening tool, but physiotherapists are generally advised to consider all prognostic (psychosocial) patient characteristics.

It is unclear to what extent screening tools or other patient-reported measurement instruments are actually being used in daily physiotherapy practice of patients with LBP in the Netherlands. Furthermore, as far as we know, no study yet focused on the underlying reasons of physiotherapists to use these instruments and their perceived added value for clinical decision-making. Better insights into the (non-) usage of measurement instruments could lead to recommendations on the most preferable instrument for a specific purpose or the need for the development of a better instrument. Finally, it is unkown whether (non-) usage of measurement instrument are related to physiotherapist characteristics. For example, it seems plausible that more experienced physiotherapists feel that they do not need such instruments but can rely on their own expertise, so they do not use any of the available screening tools.

Therefore, the aim of this study is to evaluate (i) the current use of (prognostic) measurement instruments (screening tools and questionnaires) in daily physiotherapy practice, (ii) the underlying reasons for using these instruments and (iii) the perceived influence of these instruments on the physiotherapists’ clinical decision-makingin acute LBP patients. In addition, we aim to (iv) explore whether general characteristics of physiotherapists (age, gender, working experience, e-health affinity) are related to (non-) usage of measurement instruments in acute LBP.

## Methods

### Design

Survey study among physiotherapists in primary care working with patients with LBP.

### Participants

Between November 2018 and January 2019, we invited physiotherapists from multiple LBP-related and regional networks throughout the Netherlands to participate in this survey study. Invitations were made through electronic newsletters and mailings, and we sent them one reminder. In addition, we posted an invitation for this survey on social media. We aimed at receiving a sufficient number of completed surveys to reach the (conservative) recommendation of 10 events (cases) for each predictor variable [22], which is 40 cases in our study, considering our 4 predictor variables. However, we aimed at reaching at least 80 respondents in order to provide relevant descriptive information on measurement instrument usage as well. All surveys are filled out anonymously according to the Helsinki Declaration, thereby impossible to be linked to individual respondents.

### Survey

Our survey (see Additional File 1) consisted of questions about the characteristics of physiotherapists and their use of patient-reported measurements instruments (screening tools and questionnaires) among acute LBP patients. In addition, we asked physiotherapists to offer their opinions about an ideal screening tool for acute LBP. All questions were multiple-choice questions, except for age. For the questions on currently used measurement instruments, respondents could select all available instruments for LBP patients in the Netherlands. Questions about characteristics of current use of measurement instruments applied only to the one instrument the respondent reported using most frequently.

### Analysis

First, we analysed the survey results descriptively for the total sample, using frequencies and percentages. Second, we explored the association between physiotherapist characteristics (i.e. gender, number of working years, number of LBP patients, overall e-health affinity) with measurement instrument use. For this analysis, we performed linear (for continuous outcomes measures) and logistic (for dichotomous outcome measures) regression analyses with a backwards selection. That meant that all physiotherapist characteristics were initially included in the model, after which the factor with the highest *p*-value (if higher than 0.05) was removed from the model until only factors with *p* < 0.05 were included. Outcome variables in these analyses were ‘number of measurement instrument used’ (continuous measure; our primary outcome measure), ‘underlying reason for using measurement instrument (yes/no)’ (dichotomous, for each of the given reasons), ‘perceived influence of measurement instrument on clinical decision-making’ (often or almost often vs. never, seldom or sometimes)’ (dichotomous) and, only for the most frequently used instrument, ‘usage (yes/no)’ (dichotomous). Median-split dichotomisation was performed for the determinants in these analyses, which were the following physiotherapist characteristics next to gender: physiotherapist experience (0–5 years or 6–10 years vs. 11–20 years or more than 20 years working as physiotherapist), LBP experience (0–5, 6–10 or 11–20 vs. 26–50 or more than 50 LBP patients per year) and overall self-perceived e-health affinity (not at all, low or average vs. high or very high level of being open-minded to e-health). We conducted the analysis in SPSS version 25.

## Results

A sample of around 650 physiotherapists from LBP-related and regional networks in primary care and located throughout the Netherlands received the survey between November 2018 and January 2019. In addition, an unknown number of physiotherapists viewed an invitation on social media. We received completed surveys from 85 physiotherapists (13% of the invited sample). Table 1 describes the general characteristics of this study sample.

**Table 1:** General characteristics of physiotherapists (*n* = 85)

Gender, male, n (%)	44 (52%)
Age, mean ± standard deviation	39 ± 11
Number of years working as physiotherapist: n (%)	
0–5 years	13 (15%)
6–10 years	21 (25%)
11–20 years	27 (32%)
> 20 years	24 (28%)
Number of LBP patients/year: n (%)	
0–5 patients	2 (2%)
6–10 patients	4 (5%)
11–25 patients	18 (21%)
26–50 patients	15 (18%)
> 50 patients	46 (54%)
Overall affinity with e-health: n (%)	
very high	9 (11%)
high	28 (33%)
average	31 (37%)
low	11 (13%)
not at all	5 (6%)
missing	1 (1%)

*LBP* low back pain

Almost all the respondents (98%) reported using at least one patient-reported measurement instrument for their patients with acute LBP (see Table 2). A majority of respondents reported using two instruments: the Quebec Back Pain Disability Scale (QBPDS) [23] (64% of respondents) and the STarT Back Screening Tool (SBT) [19] (61%). Other, less frequently used, instruments are the Oswestry Low Back Pain Disability Questionnaire (ODI) [24] (27%), the Patient-Specific Functional Scale (PSFS) [25] (20%), the Four Dimensional List of Complaints (4DKL) [26] (19%), the Tampa Scale for Kinesiophobia [27] (16%) and the Fear-avoidance Beliefs Questionnaire (FABQ) [28] (15%). The number of patient-reported measurement instruments used ranged between 0 and 8 with an average of 2.8. When physiotherapists had to choose the one single instrument that they use most frequently with acute LBP patients, the SBT (38%) was chosen more often than the QBPDS (31%).

**Table 2:** Usage of available measurement instrument for acute LBP (*n* = 85)

Measurement instrument used: n (%)		
No	2 (2%)	
Yes	83 (98%)	
Number of measurement instruments used: n (%)		
None	2 (2%)	
One	20 (24%)	
Two	25 (29%)	
More than two	38 (45%)	
	**All instruments used (more options possible)**	**Most frequently used instrument** **(one option)**
Specific measurement instrument used: n (%)		
QBPDS	54 (64%)	27 (32%)
SBT	52 (61%)	32 (38%)
ODI	23 (27%)	7 (8%)
PSFS	17 (20%)	11 (13%)
4DKL	16 (19%)	1 (1%)
Tampa Scale for Kinesiophobia	14 (16%)	1 (1%)
FABQ	13 (15%)	1 (1%)
CSI	9 (11%)	2 (2%)
ALBPSQ-DLV	7 (8%)	1 (1%)
IPQ	7 (8%)	0 (0%)
LAZeps	5 (6%)	0 (0%)
PCI	4 (5%)	0 (0%)
VAS or NRS for pain	4 (5%)	1 (1%)
RMDQ	2 (2%)	0 (0%)
LBPPS	1 (1%)	1 (1%)
OMPSQ	0 (0%)	0 (0%)

*4DKL* Vier Dimensionale Klachtenlijst (Four Dimensional List of Complaints);

*ALBPSQ-DLV* Acute Low Back Pain Screening Questionnaire – Dutch Language Version;

*CSI* Central Sensitization Inventory;

*FABQ* Fear-avoidance Beliefs Questionnaire;

*IPQ* Illness Perception Questionnaire;

*LAZeps* Lage rug Activiteiten Zelfvertrouwen perceptie schaal (Low back Activities Confidence perception scale);

*LBPPS* Low Back Pain Perception Scale;

*NRS* Numeric Rating Scale;

*ODI* Oswestry Low Back Pain Disability Questionnaire;

*OMPQ* Orebro Musculoskeletal Pain Screening Questionnaire;

*PCI* Pain Coping Inventory;

*QBPDS* Quebec Back Pain Disability Scale;

*RMDQ* Roland Morris Disability Questionnaire;

*SBT* STarT Back Screening Tool;

*PSFS* Patient-Specific Functional Scale;

*VAS* visual analogue scale

The measurement instruments were most often completed by the patient online (58%) or by the physiotherapist after interviewing the patient (44%) (see Table 3). Physiotherapists generally use screening tools during (or even before) intake (94%) and, in some cases, during (34%) or at the end of treatment (47%) as well. Treatment effect evaluation (53%) and symptom assessment (51%) were the most frequently reported reasons for using these instruments. More than a quarter (28%) of the respondents reported that the outcome of the instrument often or always influenced their clinical decision-making. Almost two-thirds (65%) of the respondents reported discussing the outcome of the instrument with most or all of their patients. When referring to an ‘ideal’ screening tool for acute LBP, respondents said applicability (35%) was the most preferable feature. A risk profile classification (56%) was chosen as most preferable type of outcome from a screening tool.

**Table 3:** Characteristics of measurement instrument usage for most frequently used screening tool, SBT and QBPDS

	Most frequently used measurement instrument (*n* = 83)	SBT (*n* = 32)	QBPDS (*n* = 27)
Measurement instrument completed by^a^			
patient online	48 (58%)	23 (72%)	12 (44%)
PT through interview with patient	37 (45%)	14 (44%)	11 (41%)
patient on paper	14 (17%)	7 (22%)	4 (15%)
missing	2 (3%)	0 (0%)	1 (4%)
Measurement instrument used at^a^			
start of treatment period	78 (94%)	32 (100%)	23 (85%)
end of treatment period	39 (47%)	7 (22%)	16 (59%)
during treatment period	28 (34%)	5 (16%)	11 (41%)
missing	1 (1%)	0 (0%)	1 (4%)
Underlying reason to use measurement instrument^a^			
to evaluate treatment effect	44 (53%)	9 (28%)	17 (63%)
to assess symptoms	42 (51%)	19 (59%)	13 (48%)
to estimate prognosis	29 (35%)	22 (69%)	5 (19%)
to support clinical decision-making	24 (29%)	15 (47%)	0 (0%)
to support patient education	24 (29%)	7 (22%)	7 (26%)
as required by insurance company	20 (24%)	6 (19%)	6 (22%)
as recommended in guideline	14 (17%)	6 (19%)	4 (15%)
Outcome influenced clinical decision-making			
(almost) always	3 (4%)	1 (3%)	0 (0%)
often	20 (24%)	13 (41%)	2 (7%)
sometimes	41 (49%)	13 (41%)	18 (67%)
seldom	15 (18%)	4 (12%)	5 (19%)
never	3 (4%)	1 (3%)	1 (4%)
missing	1 (1%)	0 (0%)	1 (4%)
Outcome discussed with patient			
(almost) always	27 (33%)	9 (28%)	10 (37%)
often	28 (34%)	11 (34%)	8 (30%)
sometimes	18 (22%)	6 (19%)	6 (22%)
seldom	9 (11%)	6 (19%)	2 (7%)
never	0 (0%)	0 (0%)	0 (0%)
missing	1 (%)	0 (0%)	1 (4%)
Most important feature of measurement instrument			
applicability (fast and easy)	29 (35%)	n/a	n/a
reliability/validity	27 (33%)		
interpretability	20 (24%)		
inside electronic health record	4 (5%)		
other	2 (2%)		
missing	1 (1%)		
Most preferable outcome of measurement instrument			
risk profile classification	46 (55%)		
probability	24 (29%)	n/a	n/a
yes/no prediction	12 (14%)		
missing	1 (1%)		

^a^ more than one option possible;

*QBPDS* Quebec Back Pain Disability Scale, *SBT* STarT Back Screening Tool, *PT* physiotherapist

We performed additional, explorative analyses on the two most frequently used instruments (i.e. SBT and QBPDS). All respondents (100%) reported using the SBT at the start of treatment, but only a small minority used it mid-treatment (16%) or post-treatment (22%). They most often used the SBT for prognostic purposes (69%) and its outcome influenced their clinical decision- making more often (44% reported ‘often’ or ‘always’) than the other instruments. In contrast, respondents used the QBPDS not only at the start of treatment (85%), but also mid-treatment (41%) or post-treatment (59%). The QBPDS was used primarily to evaluate treatment effect (63%) and its outcome influenced clinical decision-making less often (92% reported ‘sometimes’ or even less frequently) than the other instruments.

Male gender was found to be the only physiotherapist characteristic that is associated with the number of measurement instruments used (*p* < 0.001) (see Table 4). More specifically, male physiotherapists use on average 3.3 instruments, whereas female physiotherapists 2.0. No association was found for work experience, LBP experience or e-health affinity with number of measurement instruments. Similar results were yielded from a logistic regression model with a dichotomized outcome measure ‘low vs. high number of measurement instruments used’. In addition, none of the physiotherapist characteristics were found to be associated with perceived influence of measurement instrument on clinical decision-making or with any of the underlying reasons for using a measurement instrument.

**Table 4:** Prediction models for association between measurement instrument usage and therapist characteristics

	Number of measurement instruments			Use of SBT (yes/no)			Use of QBPDS (yes/no)		
	B	95% CI	p-value	OR	95% CI	*p*-value	OR	95% CI	*p*-value
Gender	1.3	0.6–2.0	< 0.001	n.s.			n.s.		
PT experience^a^	n.s.			n.s.			0.3	0.1–0.9	0.029
LBP experience^b^	n.s.			4.2	1.6–11.1	0.004	0.2	0.1–0.5	0.001
E-health affinity^c^	n.s.			n.s.			n.s.		

^a^ PT experience dichotomised as: ≤10 years (reference group) vs. > 10 years;

^b^ LBP experience dichotomised as: ≤50 patients/year (reference group) vs. > 50 patients/year

^c^ E-health affinity dichotomised as: no to average affinity (reference group) vs. high to very high affinity;

*B* b-coefficient;

*OR* odds ratio;

*CI* confidence interval;

*SBT* STarT Back Screening Tool;

*QBPDS* Quebec Back Pain Disability Scale;

*PT* physiotherapist

*n.s.* not significant (*p* > 0.05)

Additional, explorative regression analyses for SBT usage revealed that LBP experience is the only associated physiotherapist characteristic (OR = 4.2; *p* = 0.004), i.e. physiotherapists who see more LBP patients use the SBT more often than physiotherapists who see fewer LBP patients. For QBPDS usage, we found associations for LBP experience (OR = 0.2; *p* = 0.001) and physiotherapist experience (OR = 0.3; *p* = 0.03). However, those associations were inverse: physiotherapists who see more LBP patients or who have more years of experience as a physiotherapist use the QBPDS less often than physiotherapists with less experience.

## Discussion

This survey study of 85 physiotherapists found that nearly every respondent used one or more (prognostic) screening tool or other patient-reported questionnaire for acute LBP patients, with an average of nearly three instruments. Of these instruments, the STarT Back Screening Tool (SBT) and the Quebec Back Pain Disability Scale (QBPDS) were most frequently used for this patient group. Remarkably, despite physiotherapists’ wide acceptance of the use of (patient-reported) measurement instruments in cases of acute LBP, they reported that these instruments generally do not substantially influence their clinical decision-making.

According to our survey, Dutch physiotherapists most frequently use the QBPDS (used by 64% of physiotherapists, with 32% reporting this to be the most frequently used instrument) and the SBT (61 and 38%, respectively) with LBP patients. The QBPDS [23] is a 20-item questionnaire that is recommended in the current Dutch LBP guideline for physiotherapists [13], so it is no surprise that so many physiotherapists use it. However, the QBPDS was developed and is recommended to be used for treatment effect evaluation rather than as a screening tool. In line with this, our physiotherapists reported that treatment effect evaluation was their most important purpose for using the QBPDS in cases of acute LBP (63% of respondents), whereas only a minority of respondents (19%) used it for prognosis estimation.

Apart from the QBPDS, the SBT was the most frequently used screening tool for acute LBP patients. However, more experienced physiotherapists seem to use the SBT more often than the QBPDS. The SBT [18] is a nine-item screening tool that is widely accepted in daily practice, despite its relative newness (it was introduced in 2008 [29]). The SBT aims to classify patients into one of three risk profiles: the low-risk group should receive minimal care, the medium-risk group should receive ‘average’ physiotherapy treatment, and the high-risk group should receive more complex and intensive psychologically informed physiotherapy treatment [16]. This risk classification with treatment advice appears to correspond with the preferences of our survey respondents, who reported that a risk profile classification was the most preferable outcome for an ‘ideal’ screening tool. On the other hand, physiotherapists have criticised the risk profiles from the SBT. For example, a study by Woods and Gaskell [30] found that physiotherapists reported several perceived barriers to using the SBT, namely a *‘perceived oversimplification of the decision making process’*, an ‘*impact on professional reputations and professional development’*, ‘*risks associated with single treatment sessions*’, ‘*patient satisfaction’* and ‘*threats to patient-centered care’*. Apparently, physiotherapists have conflicting preferences regarding such screening tools.

Both the SBT and the QBPDS have recently been included in a standard set of measurement instrument for LBP patients that will be implemented in daily physiotherapy practice in the Netherlands [31]. In addition to the SBT and the QBPDS, the set of measurement instruments includes the Patient-Specific Functional Scale (PSFS) and Oswestry Low Back Pain Disability Questionnaire (ODI) (our third and fourth most frequently used instruments), as well as a Numeric Rating Scale (NRS) item for pain intensity and a Global Perceived Effect (GPE) item. According to this standard set, the SBT should be used to distinguish low-risk patients from medium- and high-risk patients. For low-risk patients, only the PSFS and NRS should be used at the start and end of treatment, and the GPE should be used at the end of treatment. For medium- and high-risk patients, all the instruments in this standard set should be used at the start of treatment, every six weeks and at the end of treatment. In addition, the GPE should be used at the end of treatment for those patients. Based on our survey, many physiotherapists are already using these instruments. If this standard set is implemented successfully, its usage will become more standardised and uniform. Ideally, this could be achieved by recommending this set in a physiotherapy guideline for LBP.

We expected that physiotherapists with more work experience might use screening tools and questionnaires less frequently than physiotherapists with less experience, as this first group would rely more on their own expertise. However, we did not find such an association, except for the finding that more experienced physiotherapists tend to choose the SBT rather than the QBPDS as a screening tool for their patients with acute LBP. This decision seems to be in line with the main purpose of the SBT (i.e. prognosis) and QBPDS (i.e. treatment evaluation). Male gender was the only physiotherapist characteristic for which we found an association with using a larger number of instruments for acute LBP, but this findings needs to be interpreted with caution due to the explorative character of this analysis. We do not know why male physiotherapists reported to use more instruments compared to female physiotherapists.

Our findings suggest that physiotherapists are not convinced of the benefits of the currently available (prognostic) measurement instruments for daily physiotherapy practice, because their outcomes did not affect clinical decision-making about most patients. One reason could be that none of the existing instruments (including screening tools) are yet able to adequately predict the course of LBP symptoms, so they do not provide any relevant prognostic information for the physiotherapist. Despite the enormous effort put into prognostic LBP research, LBP screening tools all perform poorly in identifying those patients at higher risk for chronic LBP [32]. Even for the SBT, the accuracy of prediction of outcome of patients with LBP was low [33]. This finding suggests that new research may need to focus on developing a better measurement instrument (e.g., screening tool) that do fits the needs of physiotherapists. In an ongoing study, we aim to develop and subsequently validate such a new screening tool that includes an algorithm that adequately predicts the chance of recovery within three months, in patients with acute LBP. In the development phase of this tool, the (practical) preferences from physiotherapists are considered essential, in order to have large impact on daily practice. A second reason for scepticism about the added value of measurement instruments could be that physiotherapists prefer to rely on their own expertise for their clinical decision-making, although their predictive ability is debateable [33]. A third reason might be that physiotherapists only use measurement instruments because of perceived obligations from external parties, like insurance companies (reported by 24% of respondents) or clinical guidelines (17%). If they only use these tools because external parties require them to, physiotherapists may not intend to use them in treatment decisions. Finally, physiotherapists may generally provide a ‘one-size-fits-all’ treatment for patients with LBP [14], so the outcome of any measurement instrument will not affect their clinical decisions.

Regardless of the underlying reasons for using measurement instruments, our study may suggest that measurement instruments (e.g., screening tools) should be further optimised to have more added value for daily practice. Based on the preferences reported in our survey, such an instrument should be very easy and quick to apply on one hand, but also highly reliable and valid on the other. Furthermore, the instrument’s impact on clinical decision-making might be optimised if it is fully integrated into the electronic health record. Ideally, this would give physiotherapists clear treatment advice in a highly intuitive way, although such treatment advice is quite controversial among some clinicians because it could threaten their professional role. In addition, our study suggests that physiotherapists should be more critical about which instruments to use and for which purpose. It seems that many instruments are being applied standardly, but without integrating the information retrieved from the instrument adequately in their clinical decision making. Thereby, the potential of measurement instruments is not fully utilized.

Our study had some limitations that should be acknowledged. First, our findings are based on the results of a survey with a 13% response rate, without a sample size calculation. Due to our response rate, we cannot be sure that these findings are representative for all physiotherapists working with LBP in the Netherlands. It can be assumed that physiotherapists with an interest in LBP were more willing to participate in our survey study. However, non-response in survey studies does not seem to result in major response bias, even if non-response is non-random (e.g. related to respondents’ interests) [34, 35]. In addition, the general characteristics of our respondents seem to correspond with those of Dutch physiotherapists (i.e. 52% male in our study vs. 41% in the Dutch physiotherapy population; mean age of 39 years in our study vs. 43 years in the Dutch physiotherapy population [36]). Moreover, sample size calculations are not considered essential in survey studies like ours. Second, we initially aimed to provide insight into the use or non-use of prognostic screening tools in particular, but decided to include all patient-reported measurement instruments that are currently being used with LBP patients, most of which are not screening tools. We decided to do that to avoid any misunderstanding, as we expected that physiotherapists may not be aware of the difference between a prognostic screening tool and any other measurement instrument. Thereby, our study results apply to measurement instruments in LBP in general, instead of prognostic instruments specifically.

## Conclusion

Our survey indicates that patient-reported measurement instruments are widely used by physiotherapists in cases of acute LBP, but mostly for non-prognostic reasons, such as treatment effect evaluation or symptom assessment. Moreover, current instruments seem to have limited added value for clinical decision-making, according to physiotherapists. Possibly, a new measurement instrument (e.g., screening tool) needs to be developed that does fit the physiotherapist’s needs and preferences. Our findings also suggest that physiotherapist may need to be more critical about which measurement instrument they use and for which purpose.

## Supplementary information

**Additional file 1.** Survey

## Data Availability

The dataset supporting the conclusions of this article is available in the open dataset repository of our institute (DANS Easy; 10.17026/dans-zy2-tjr8).

## Abbreviations

4DKL: Vier Dimensionale Klachtenlijst (Four Dimensional List of Complaints)
GPE: Global Perceived Effect
LBP: Low back pain
NRS: Numeric Rating Scale
ODI: Oswestry Low Back Pain Disability Questionnaire
OMPQ: Orebro Musculoskeletal Pain Screening Questionnaire
PSFS: Patient-Specific Functional Scale
QBPDS: Quebec Back Pain Disability Scale
SBT: STarT Back Screening Tool
VAS: Visual analogue scale
