# Supplementary material for: Use and perceived added value of patient-reported measurement instruments by physiotherapists treating acute low back pain: a survey study among Dutch physiotherapists
Source: BMC Musculoskelet Disord. 2020 Feb 24;21:120. doi: 10.1186/s12891-020-3132-9 (PMC7041183; doi:10.1186/s12891-020-3132-9)
Supplement: Supplementary file 1 — Additional file 1. Survey [file 12891_2020_3132_MOESM1_ESM.docx]

***Additional file 1 : Survey***

*We aim to develop an easy applicable screening tool for physiotherapists to estimate the risk for chronicity in patients with acute nonspecific low back pain (episode less than 1 month). This app-based and/or web-based screening tool should be able to provide a prediction for the presence/absence of low back pain after 3 months, based on patient characteristics from baseline and/or the first weeks of treatment. To develop such an easy applicable tool, we first want to explore your current application of available screening tools in daily practice and your preferences for these tool. Therefore, we would like you to complete this short survey, which will take approximately 10 minutes of your time. Thanking you in advance.*

1. Male / female
2. Age : ……….… years
3. How many years are you working as physiotherapist?
   - 0-5 years
   - 6-10 years
   - 11-20 years
   - More than 20 years
4. How many patients with low back pain consult you on average per year?
   - 0-5 patients with low back pain
   - 6-10 patients with low back pain
   - 11-25 patients with low back pain
   - 26-50 patients with low back pain
   - More than 50 patients with low back pain
5. Which screening tool or other measurement instrument do you apply in patients with acute low back pain? (more than one answer possible)
   - VierDimensionale Klachtenlijst
   - Acute Low Back Pain Screening Questionnaire-Dutch Language Version
   - Central Sensitization Inventory
   - Fear-avoidance Beliefs Questionnaire
   - Illness Perception Questionnaire
   - Lage rug Activiteiten Zelfvertrouwen perceptie schaal
   - Low Back Pain Perception Scale
   - Oswestry Low Back Pain Disability Questionnaire
   - Örebro Musculoskeletal Pain Screening Questionnaire
   - Pain Coping Inventory
   - Quebec Back Pain Disability Scale
   - Roland Morris Disability Questionnaire
   - STarT Back tool
   - Tampa schaal for Kinesiofobie
   - Other, namely………………………………………………
6. Which screening tool or other measurement instrument do you apply most frequently in patients with acute low back pain? (one answer possible; the following 5 questions will relate to this instrument)
   - VierDimensionale Klachtenlijst
   - Acute Low Back Pain Screening Questionnaire-Dutch Language Version
   - Central Sensitization Inventory
   - Fear-avoidance Beliefs Questionnaire
   - Illness Perception Questionnaire
   - Lage rug Activiteiten Zelfvertrouwen perceptie schaal
   - Low Back Pain Perception Scale
   - Oswestry Low Back Pain Disability Questionnaire
   - Örebro Musculoskeletal Pain Screening Questionnaire
   - Pain Coping Inventory
   - Quebec Back Pain Disability Scale
   - Roland Morris Disability Questionnaire
   - STarT Back tool
   - Tampa schaal for Kinesiofobie
   - Other, namely………………………………………………
7. How is this instrument completed? (more than one answer possible)

- By patient on paper
- By patient online
- By myself (through interviewing)
- Other, namely……………..

1. At what time-point do you apply this instrument? (more than one answer possible)

- At start of treatment period (during/before intake)
- During treatment period
- At end of treatment period
- Other time-point, namely………………………..

1. For which underlying reason do you apply this instrument in patients with acute low back pain? (more than one answer possible)

- Diagnostic: to assess the severity and/or type of symptoms
- Prognostic: to estimate the likely course of symptoms
- Evaluative: pre-/post-treatment measurement to evaluate treatment effect
- To support clinical decision making
- To support patient education
- Because recommended in guideline
- Because obligated by insurance company
- Other reason, namely………………………

1. To what degree influences this instrument your clinical decision making?
   - (almost) always
   - often
   - sometimes
   - seldom
   - never
2. To what degree do you discuss the outcome of this instrument with your patient?
   - (almost) always
   - often
   - sometimes
   - seldom
   - never
3. What is your most preferable feature of an ideal screening tool in general, to apply this in daily practice?
   - Reliability/validity (to have a precise prediction of the real course)
   - Applicability (to apply fast and easy)
   - Interpretability (to easily understand the outcome and discuss this with my patient)
   - Embedded within my electronic health record (therefore no additional webste, app, or program is needed)
   - Other feature, namely …………………………….
4. What would be your most preferable outcome of a screening tool to estimate the course of pain in patients with acute low back pain?
   - A prediction (yes/no) of the presence of pain after 3 months
   - A chance or likelihood (%) of pain present after 3 months
   - A risk profile classification
   - Other outcome, namely………………………..
5. The screening tool that will be developed will be a digital (e-health) tool, namely an app and/or website that can be used by you or your patient. To what degree are you generally open-minded for the application of e-health tools in daily practice, like blended care, apps and wearables?
   - very high
   - high
   - average
   - low
   - not at all
